# PIT-1/SF-1-positive pituitary tumors in patients with acromegaly: transcriptomic perspective

**DOI:** 10.1186/s40478-025-02091-z

**Published:** 2025-08-14

**Authors:** Julia Rymuza, Qilin Zhang, Mateusz Bujko

**Affiliations:** 1 Department of Molecular and Translational Oncology, Maria Sklodowska-Curie National Research Institute of Oncology, Warsaw, 02-781 Poland; 2 Department of Neurosurgery, Huashan Hospital, Shanghai Medical College, Fudan University, Shanghai, 200040 China; 3 National Center for Neurological Disorders, Shanghai Medical College, Huashan Hospital, Fudan University, Shanghai, 200040 China

**Keywords:** Pituitary neoplasms, Growth hormone-secreting pituitary adenoma, Gene expression profiling, Single-cell gene expression analysis, Transcription factors

## Abstract

Neuroendocrine pituitary tumors (PitNETs) are classified based on clinical manifestation and expression of pituitary cell lineage-specific transcription factors (TFs) and hormones. A subtype of tumors in patients with acromegaly was found to express PIT-1 and SF-1 TFs, two markers of distinct pituitary cell lineages. These tumors have been described as multilineage or “somatogonadotoph” tumors. The aim of our study was to clarify their identity and cell type origin using extensive transcriptomic analysis. For this purpose, we analyzed the RNA sequencing (RNAseq) data from 546 PitNETs (including 193 tumors of patient with acromegaly) and single cell RNAseq data from somatotroph and gonadotroph tumors and normal pituitary tissue. Somatrotroph PitNETs co-expressing PIT-1 and SF-1 TFs were identified in each of the analyzed RNAseq dataset. Their transcriptomic profile and pituitary TF activity closely resembled those of other somatotroph tumors, while differing substantially from gonadotroph PitNETs, though they retained *NR5A1* (SF-1) activity and expressed some SF-1-regulated genes (e.g., *LHB* and *GNHRH*). Notably, SF-1 appeared to regulate a slightly different set of genes in double positive somatotroph PitNETs and gonadotroph tumors. Analysis of scRNAseq data revealed a subcluster of normal gonadotroph cells expressing *POU1F1* (PIT-1), but tumor cells from PIT-1/SF-1 PitNETs did not resemble this normal gonadotroph cell subtype. Genes expression profiles of three subtypes of somatotroph tumors were distinguished through analyses of both bulk- and scRNAseq data. From transcriptomic perspective — based on gene co-regulation and pituitary TF activity — PitNETs of patients with acromegaly that co-express PIT-1 and SF-1 represent a subtype of PIT-1 lineage tumors, and the molecular data do not support classifying them as multilineage tumors.

**Supplementary Information:**

The online version contains supplementary material available at 10.1186/s40478-025-02091-z.

## Introduction

Neuroendocrine pituitary tumors (PitNETs), also known as pituitary adenomas, are among the most common intracranial tumors in adults. They are a heterogenous group of tumors, that originate from distinct pituitary secretory cell types. Pituitary tumors are classified according to the recommendations of the fifth edition of the WHO Classification of Endocrine and Neuroendocrine Tumors [1]. This classification is based on the paradigm that mature pituitary cells, which secrete specified tropic hormones, arise from common progenitor cells through a differentiation process orchestrated by a set of specific transcription factors (TFs). The key transcription factors in the development of mature corticotroph and gonadotroph pituitary lineages are TPIT (encoded by the *TBX19* gene) and SF-1 (*NR5A1*), respectively. In turn, the transcription factor PIT-1 (*PUO1F1*) is crucial for the maturation of somatotroph, lactotroph and tyrotroph cells. Thus, somatotroph pituitary tumors (sPitNETs) are, by definition, those that express the PIT-1 transcription factor and produce growth hormone (GH). In addition to somatotroph tumors, other subtypes of GH- and PIT-1-positive tumors cause acromegaly: mammosomatotroph tumors and mature plurihormonal tumors (characterized by the expression of GH along with additional hormones such as prolactin (PRL) and thyrotropic hormone), mixed somatolactotroph tumors (comprising two distinct populations of somatotroph and lactotroph tumor cells), and poorly differentiated PIT-1–positive tumors [2, 3].

According to recently published data, somatotroph pituitary tumors that cause acromegaly fall into three distinct molecular subtypes [4–7]. These subtypes differ in gene expression, DNA methylation, and DNA copy number profiles, as well as in the frequency of *GNAS* mutations, granularity pattern on electron microscopy, the proportion of invasive tumors and tumor size [4–7]. In our genes expression analyses, we found that one of these three subtypes (referred to as Subtype 1 in our previous report [7]) is characterized by the expression of *NR5A1* (SF-1), a marker of gonadotroph PitNETs. Tumors co-expressing both PIT-1 and SF-1 transcription factors have occasionally been reported by others over the years [8–14] and have been highlighted as a matter of special interest. These PIT-1/SF-1 positive sPitNETs differ in several aspects from other acromegaly-associated PitNETs lacking SF-1 expression: they are densely granulated adenomas without *GNAS* mutations, with ectopic *GIPR* expression, a distinctive DNA methylation profile, and high level of genomic instability [4, 6, 7].

Recent discussions about PIT-1/SF-1 tumors have been marked by confusion, as these two TFs are considered markers of separate pituitary linages according to the official classification. Such tumors were commonly referred to as multilineage pituitary tumors [15, 16], gonado/somatotroph tumors [6], somatogonadotroph [17], or plurihormonal tumors [8, 10]. In this article we aimed to clarify the identity of double positive PIT-1/SF-1 tumors through an in-depth analysis combining our in-house generated and publicly available transcriptomic data on pituitary tumors, as well as published single-cell RNA sequencing (scRNAseq) data from both PitNETs and normal pituitary tissue. The main questions we addressed were: (i) are PIT-1/SF-1 tumors more closely related to gonadotroph or somatotroph PitNETs?, (ii) does experimental evidence justify classifying them as multilineage?, (iii) are there normal counterparts of double-positive PIT-1/SF-1 cells in the pituitary?, (iv) what distinguishes PIT-1/SF-1 tumors from other PIT-1-positive somatotroph tumors at the gene expression level?. We believe that transcriptomic perspective — particularly one focusing on the transcription factor activity provides insights aligned with the principles underlying the official WHO classification of pituitary tumors.

## Methods

### Bulk RNASeq data

We used RNAseq data previously generated by our research groups (HRA003588 [18] and E-MTAB-11889 [7]). Through a literature review, we also identified three additional datasets containing bulk transcriptomic data of PitNETs: E-MTAB-7768 [14], GSE213527 [19], GSE209903 [20]. Depending on the available data, we used either Salmon [21] to map reads to the human genome (version hg38) and taximport [22] to extract raw counts from the reads, or featuresCounts [23] to count reads from already mapped reads. For analysis, we included genes detected in all studies that had more than 10 reads in over 25% of samples. The combined dataset was then variance stabilized transformed (VST) using DESeq2 [24], and batch effects were removed with the removeBatchEffect function from the limma package [25]. Differentially expressed genes were identified using linear model applied to the transformed, batch corrected data, with *p*-values adjusted for false discovery rate (FDR).

To identify previously established transcriptomic subtypes of sPitNETs in the analyzed cohort, we focused on samples which were obtained from patients diagnosed with acromegaly, mixed acromegaly and hyperprolactinemia, or with serum IGF-1 levels above 400 ng/mL. We clustered samples using k-means clustering, hierarchical clustering, and a Gaussian Mixture Model. Samples were assigned to a cluster based on the most frequent (consensus) assignment among the three clustering methods. For samples with discordant results between the algorithms, we assigned them based on previously established marker genes for sPitNET subtypes [7].

To further investigate expression patterns, we used WGCNA [26] to identify modules of highly correlated genes and relate them to selected sample characteristics. We also computed regulons —groups of genes co-regulated by a specific TF — using the RTN package [27]. For TFs with documented function in pituitary (listed in Supplementary Table S1), we calculated their activity in the samples. Finally, we performed a two-tailed GSEA to test whether differentially expressed genes (DEGs) were enriched with identified regulons [28, 29].

### Single-cell RNAseq data

We utilized the previously generated scRNAseq data (PRJCA009690 [30]), along with other publicly available single-cell transcriptomes of both adult pituitary gland (APG) and PitNETs: GSE208108 [31]; HRA003110 [32]; HRA003483 [33]; PRJNA1137596 [34], as well as fetal pituitary (FPG) GSE142653 [35]. Raw reads were processed with alvin-fry [36] through simpleleaf framework using GRCh38 genome assembly. All datasets were further analyzed using the Seurat package (v5.1.0) [37]. Low quality cells were filtered based on *nFeature_RNA*, *nCount_RNA*, and *percent.mt*. Doublets were detected using scDblFinder [38], and only singlets were retained for further analysis. Red blood cells were identified by calculating an RBC score, using *HBB*, *HBA1*, *HBG2*, *HBA2* as marker genes. Highly variable features were selected, and the filtered count matrix was scaled and subjected to principal component analysis (PCA). Depending on data complexity, datasets were integrated using either CCA [39] or FastMNN [40] algorithms. Cell-type clusters were identified based on the expression of classical marker genes [30, 31]. To assess the similarity of tumors to normal pituitary cell types, we applied AddModuleScore using the top 20 DEGs specific to each cluster from the APG dataset.

We used Slingshot [41] and tradeSeq [42] to construct lineage trajectory of the integrated APG and FPG gonadotroph cells and to examine gene expression along pseudotime. For integrated data from APG and somatotroph tumors, we use Monocle3 [43] to calculate pseudotime-related trajectory.

We used decoupleR [44] to construct pseudobulks of somatotroph cells in each sample. DEGs between somatotroph cells from each subtype and the APG were identified using pyDESeq2 [45]. For the resulting gene sets, we performed gene set enrichment analysis (GSEA) with decoupleR, using the KEGG database [46].

To compare the regulons of key TFs between somatotroph cells from Subtype 1 and gonadotrhop cells from gonadotrhop tumors, we used hdWGCNA [47]. For each TF, we selected genes with a regulatory score greater than 0.75.

We inferred copy number (CN) profiles for each sample using CopyKAT [48], with somatotroph cells from the APG serving as the reference (normal) cells. For each sample, we calculated CN profiles as the average CN ratio for each chromosome arm. Each cell was then assigned to a stability cluster based on an aneuploidy score (AS), defined as a number of chromosome arms with an average CN ratio greater than 0.1, after normalization by subtracting CN ratio of chromosome 22 to address previously reported shortcomings of only depth-based CN calculations [4]. Cells with an AS > 2were designated as *unstable*, while those with a deletion of chromosome 11 and fewer than 4 amplifications of chromosomal arms were classified as *del11*.

## Results

### Clustering of pituitary tumor samples

**Fig. 1:**
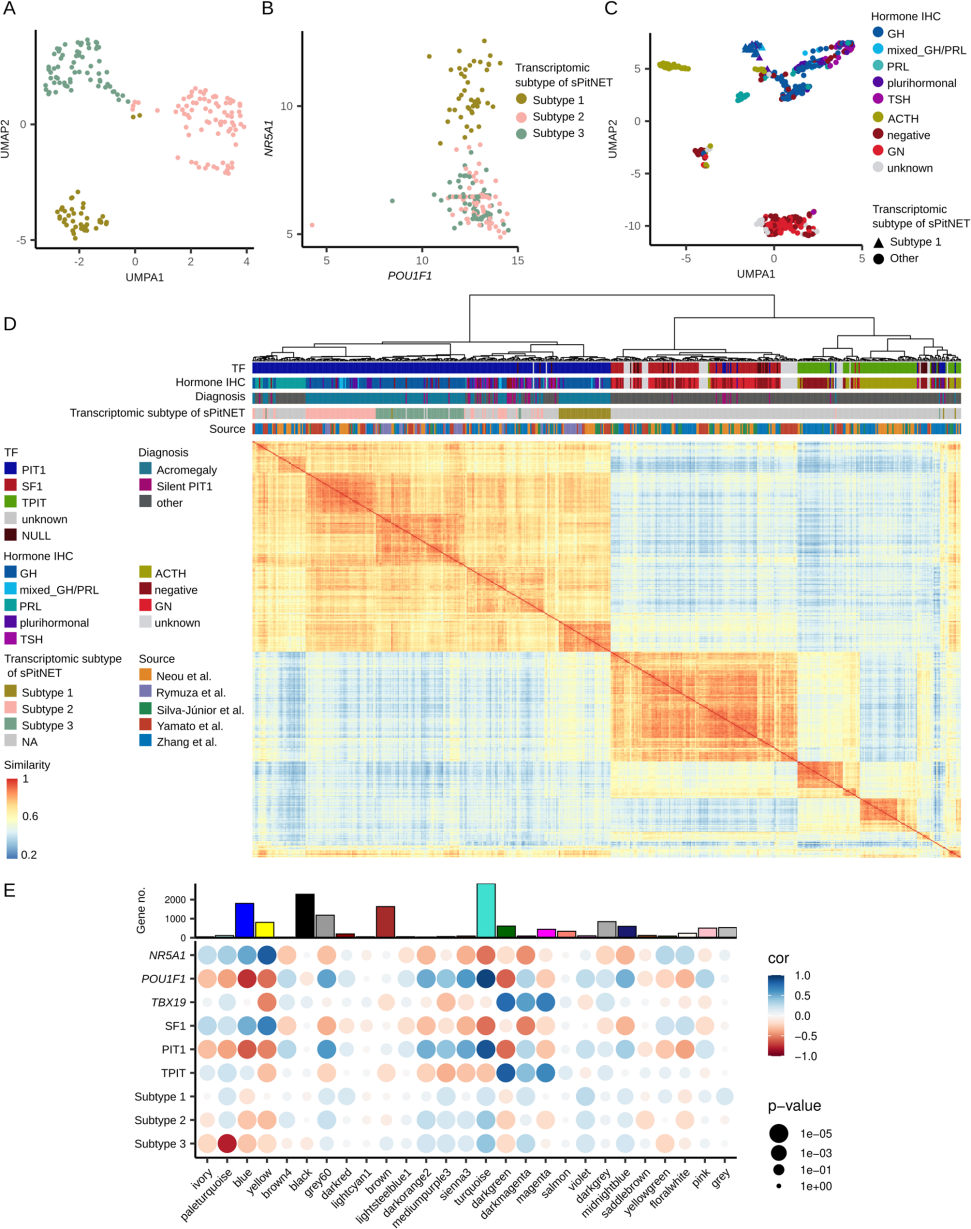
Transcriptomic profiles of acromegaly-associated pituitary tumors and the entire PitNETs cohort. (**A**) UMAP of acromegaly-associated tumors, colored by assigned transcriptomic subtypes. (**B**) Expression of *POU1F1* and *NR5A1* in acromegaly-associated tumors samples, colored by transcriptomic subtype. (**C**) UMAP of the entire PitNET study cohort, labeled according to hormone staining by immunohistochemistry (IHC), with Subtype 1 acromegaly-associated tumor samples highlighted. (**D**) Hierarchical clustering and similarity matrix of transcriptomes of the entire study cohort, summarizing molecular, histological, and clinical characteristics. (**E**) Weighted Gene Co-expression Network Analysis (WGCNA) results, showing correlation (cor) between gene modules and specific tumor features

Three molecular subtypes of somatotroph tumors causing acromegaly have been identified in previous studies. Therefore, we began our analysis by assigning each sample in the study cohort —comprising of 193 acromegaly-causing tumors— to one of these subtypes. Dimensionality reduction of transcriptomic data using UMAP revealed a clustering of tumors obtained from patients with acromegaly into three groups (Fig. 1A). Based on consensus of three clustering algorithms, 98% of the samples was assigned to one of these groups. Each group was identified as one of the previously characterized subtypes (subtypes 1, 2 and 3) [4, 6, 7], based on the presence of samples from previous studies with known transcriptome subtype assignment. Three samples (1.5% of all acromegaly-associated PitNETs) could not be confidently assigned due to lack of consensus between the three algorithms; these were manually classified based on previously proposed marker genes [7] (Supplementary Fig. S1). Altogether, this allowed us to annotate each acromegaly-associated tumor sample in the study cohort as one of the known somatotroph subtypes. Subtype 1, previously described as composed of densely granulated SF-1/PIT-1 double-positive tumors, was represented by 43/193 (22%) of samples. Subtype 2, previously described as enriched for *GNAS*-mutated densely granulated tumors, included 83/193 samples (43%). Subtype 3, known to be enriched for sparsely granulated somatotroph PitNETs, accounted for 67/193 samples (35%). Consistent with earlier findings, co-expression of *POU1F1* (PIT-1 encoding gene) and *NR5A1* (gene coding SF-1 protein) was clearly observed in the tumors of Subtype 1 (Fig. 1B). All these tumors were originally diagnosed as GH-omas or plurihirmonal tumors co-expressing GH and PRL, with one case of mixed GH/PRL and one of unknown diagnosis.

Having characterized the acromegaly-associated tumors, we next performed unsupervised clustering of all the PitNETs in the study cohort (*n* = 546) (Supplementary Table S2). We paid particular attention to the similarity of double-positive *POU1F1*-and *NR5A1*-expressing tumors to the other histological types. The samples clustered according to the expression of lineage-specific TFs into three distinct groups: PIT-1, TPIT, and SF-1 tumors (Fig. 1C). Among the tumors previously described as negative for TF expression (null cell tumors), most (13/24) clustered with SF-1 group; 8/24 clustered with TPIT samples, and 3/24 with PIT-1 cluster. The combined cohort also included 45 non-functioning PitNETs without reported immunohistochemical staining status (i.e. no information on hormone nor TF expression). These samples predominantly clustered with the SF-1 group (32 samples), while 11 grouped with TPIT tumors and two with PIT-1. Importantly, all the acromegaly-associated Subtype 1 tumors (co-expressing *POU1F1* and *NR5A1*) formed a distinct cluster located closer to the PIT-1 group than to SF-1 tumors, reflecting their much higher similarity to PIT-1-positive than gonadotroph PitNETs. This clustering pattern, obtained with UMAP, was confirmed with hierarchical clustering and similarity matrix analysis (Fig. 1D). The dendrogram illustrating sample similarities revealed three major branches corresponding to PIT-1, SF-1, and TPIT tumors. With the exception of three samples, all double-positive PIT-1/SF-1 tumors grouped as a separate cluster within PIT-1 branch. Notably, none of these tumors clustered within the SF-1 (gonadotroph) branch.

### Genes co-expression analysis

Given the much higher similarity of double-positive PIT-1/SF-1 tumors to other PIT-1 lineage tumors than to SF-1 gonadotroph tumors, we next examined whether their gene expression profiles further support their pituitary lineage identity. We performed a Weighted Gene Co-expression Network Analysis (WGCNA) of the RNAseq data from the entire study cohort to construct a gene co-expression network and identify groups of co-expressed genes. This analysis revealed 27 modules of co-expressed genes, each labeled with a distinct color (Fig. 1E). We assessed the association of each module with the expression of genes encoding for lineage-specific TFs (*NR5A1*, *POU1F1*, and *TBX19*), as well as with tumor subtypes: SF-1**–**positive gonadotrophs, TPIT-positive corticotrophs, general PIT-1 lineage tumors (including the *POU1F1/NR5A1*-expressing ones) and the three transcriptomic subtypes of acromegaly-associated tumors (Fig. 1E). We observed that the main histological subtypes, defined by the expression of lineage-specific TFs, showed similar associations with the WGCNA modules as did the expression of their respective TFs: PIT-1 tumors aligned with *POU1F1*, SF-1 gonadotroph tumors aligned with *NR5A1*, and TPIT corticotroph tumors aligned with *TBX19* expression. All three subtypes of acromegaly–associated tumors displayed clear similarity to PIT1-lineage tumors and to *POU1F1* expression in their WGCNA module associations (Fig. 1E).

Interestingly, the Subtype 1 double-positive tumors resembled SF-1-positive goadotropinomas in their relationship to only a single module (the grey module), whereas for the remaining modules their patterns were more consistent with the PIT-1 lineage.

In summary, the unsupervised gene co-expression analysis confirmed the findings from sample clustering: the double-positive Subtype 1 tumors are much more closely related to other PIT1-lineage tumors than that to SF-1-positive gonadotroph tumors. Notably, the gene co-expression patterns revealed very little similarity between PIT-1/SF-1–positive and SF-1–positive tumors.

### Regulon analysis

Transcriptomic data enables the assessment of TF activity. Since pituitary lineage-specific TFs are central to the official PitNETs classification, and a number of TFs involved in pituitary development have been identified, we considered regulon analysis to be particularly informative.

**Fig. 2:**
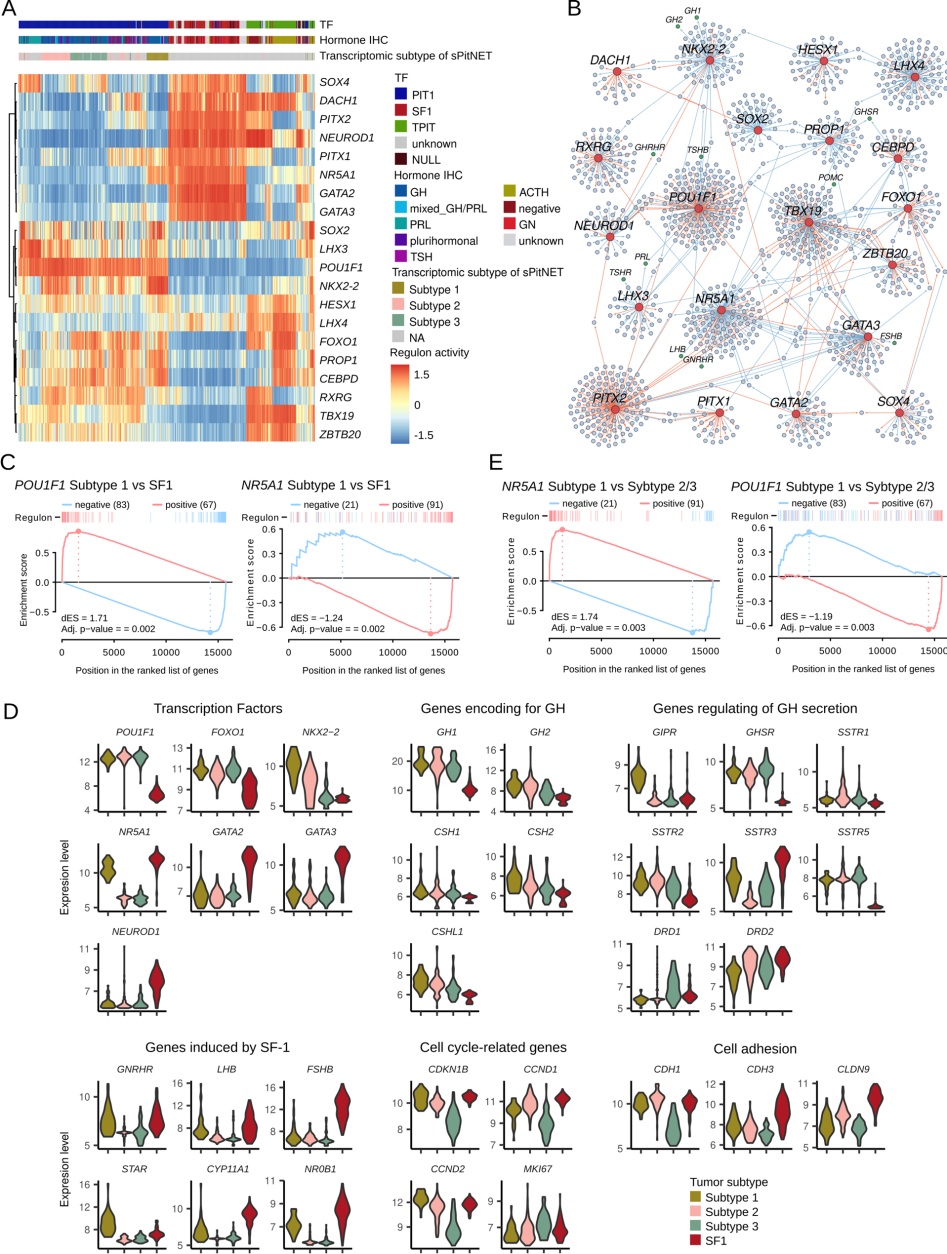
Result of regulon analysis. (**A**) Regulon activity of selected transcription factors (TF) orchestrating the differentiation of pituitary secretory cells lineages in PitNET samples, along with molecular and histological characteristics. (**B**) Network of genes regulated by selected TFs. (**C**) Gene Set Enrichment Analysis (GSEA) results showing enrichment of differentially expressed genes (DEGs) between Subtype 1 (PIT-1/SF-1-positive) somatotroph tumors and gonadotroph tumors with the regulons of *POU1F1* and *NR5A1*. (**D**) Expression levels of selected DEGs. (**E**) GSEA results showing enrichment of selected DEGs between Subtype 1 (PIT-1/SF-1-positive) somatotroph tumors and Subtype 2/3 somatotroph tumors with the regulons of *POU1F1* and *NR5A1*

We identified regulons — sets of genes regulated by a given TF — for the entire dataset based on mutual information. We then computed the activity of all the known TFs in each tumor sample. Based on the literature data, we selected 25 TFs known to be important for anterior pituitary cell differentiation and function. Of these, regulons were successfully identified for 20 TFs (five TFs were excluded because their regulon small size did not meet default thresholds ) (Supplementary Table S3). This analysis revealed regulon patterns characteristic for the major PitNETs histological subtypes, with distinct TF activity signatures in PIT-1 linage tumors, SF-1–positive gonadotropinomas, and TPIT–positive corticotropinomas (Fig. 2A). Interestingly, each TF predominantly regulated largely distinct set of genes, with relatively few genes shared by more than one TF (Fig. 2B). Of particular interest was the difference in TF activity between PIT-1– and SF-1–positive PitNETs. Double-positive (PIT-1/SF-1) acromegaly-associated tumors exhibited a TFs activity profile typical of PIT-1–positive rather than SF-1–positive tumors, except for *NR5A1*, whose activity was shared by double-positive and gonadotroph PitNETs. Other TFs specific for gonadotroph tumors, such as *GATA2*, *GATA3*, were inactive in double-positive (Subtype 1) somatotroph tumors. These differences in TF activity across subtypes of somatotroph and gonadotroph tumors were consistent with differences in both TF expression levels and in expression of the genes involved in pituitary secretory function (Fig. 2A).

We complemented the regulon analysis with a differential gene expression analysis. Differentially expressed genes (DEGs) between double-positive Subtype 1 acromegaly-associated tumors and gonadotroph SF-1 tumors were identified (Supplementary Table S4). Next, we analyzed the enrichment of DEGs with the *POU1F1* and *NR5A1* regulons using GSEA. We observed that the DEGs are strongly enriched for genes regulated by *POU1F1*, but also show enrichement for the *NR5A1* regulon. Notably, most genes transcriptionally activated by *NR5A1*, according to the inferred regulon, exhibit lower expression in double-positive sPitNETs compared to gnadotroph tumors (Fig. 2C). This suggests that *NR5A1* regulates a different set of target genes in gonadotroph and Subtype 1 acromegaly-associated tumors (Fig. 2C). Indeed, several well-known *NR5A1*-depenedent, experimentally validated genes — such as *CYP11A1*,* NR0B1*, and *FSHB* — are expressed at lower levels in double-positive sPitNETs than gonadotroph tumors, while the others — such as *GNRHR* and *LHB* —show comparable expression (Fig. 2). Some *NR5A1*-regulated genes, such as *STAR*, have higher expression in Subtype 1 somatotroph than in gonadotroph PitNETs.

We also compared Subtype 1 somatotroph tumors to the other acromegaly-associated tumors (combined Subtypes 2 and 3) and examined the enrichment of DEGs with *NR5A1* and *POU1F1* regulons (Supplementary Table S4). The DEGs were strongly enriched for *NR5A1*-regulated genes, whereas enrichment for *POU1F1*-regulated was much weaker (Fig. 2E). This finding confirms the specificity of *NR5A1* regulation in Subtype 1 tumors and suggests only a slightly altered activity pattern of *POU1F1*.

Furthermore, we used the regulon data to identify TFs most specific to double-positive PIT-1/SF-1 tumors compared to the rest of the PitNETs (Supplementary Table S5). This analysis revealed that *NKX2-2* activity was the most specific (AUC 0.98), supporting previous observation that this TF may serve as a reliable marker for this particular transcriptomic subtype [49]. Interestingly, genes characteristic of somatotroph cells — such as *GH1*, *GH2*, and *GHRHR* — were included in the *NKX2-2* regulon (Fig. 2B).

### Analysis of scRNAseq data from normal pituitary to identify normal *NR5A1*/*POU1F1* double- positive cells

**Fig. 3:**
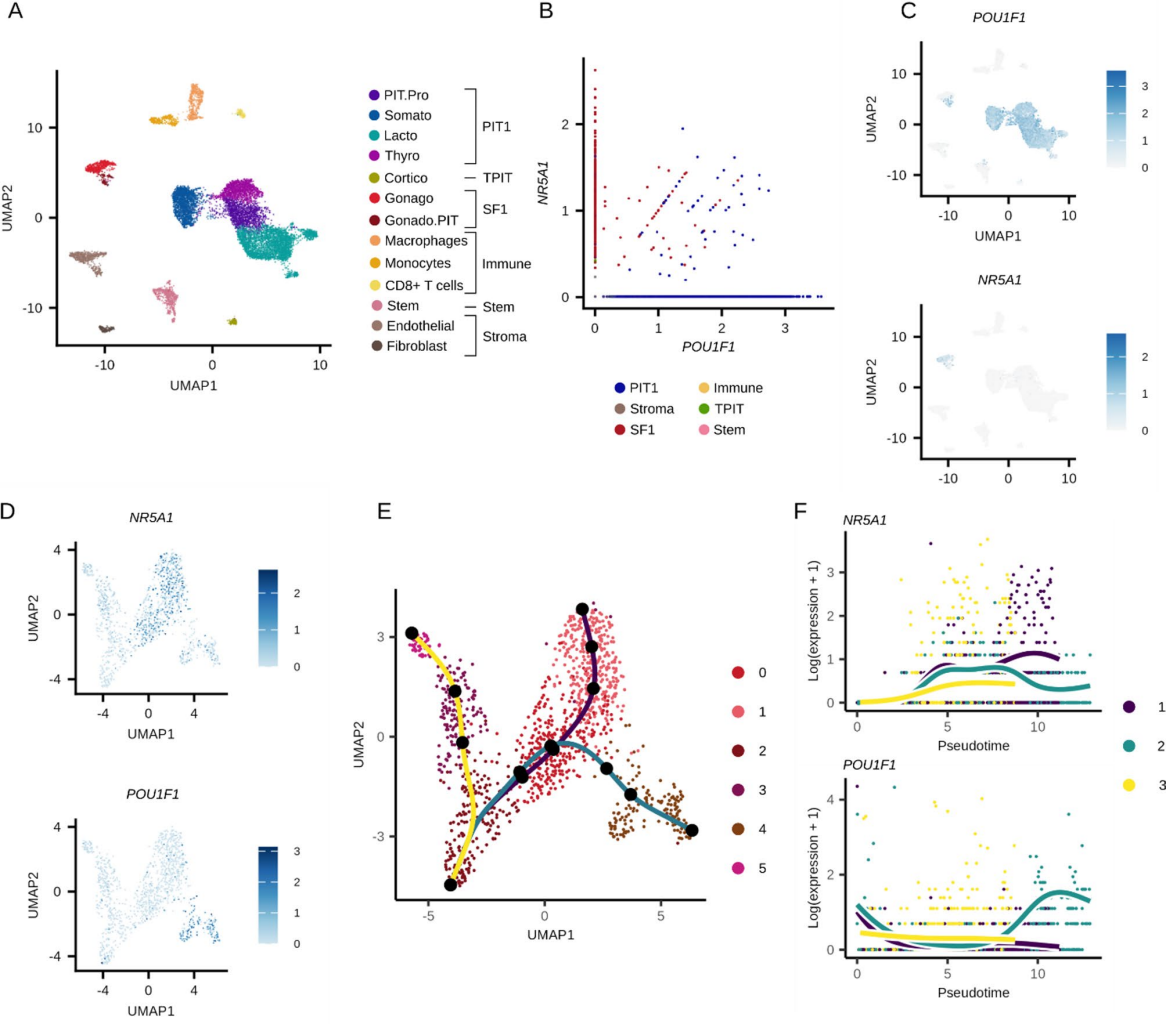
Singel-cell RNAseq analysis of adult pituitary gland (APG). (**A**) UMAP of cells from APG samples with assigned cell types. (**B**) Co-expression of *POU1F1* and *NR5A1* in APG cells, colored according to main clusters corresponding to specific cell subtypes. (**C**) Expression of *POU1F1* and *NR5A1* in APG cells. (**D**) Expression of *POU1F1* and *NR5A1* in gonadotroph cells from integrated scRNAseq data of APG and fetal pituitary. (**E**) Clustering and differentiation trajectory of gonadotroph cells from integrated APG and fetal pituitary dataset. (**F**) Expression of *POU1F1* and *NR5A1* along the differentiation trajectory of gonadotroph cells in the APG and fetal pituitary integrated dataset

We analyzed available single-cell RNAseq data from 10 samples of normal adult pituitary gland (APG). Unsupervised clustering detected 19 clusters, which were classified into 12 cell types based on marker gene expression and cluster similarity: four PIT1 lineage (PIT1.Pro, Somato, Lacto, Thyro), two SF1 lineage (Gonado, Gonado.PIT), one TPIT lineage (Cortico), three immune cell types (Macrophages, Monocytes, CD8 + T cells), one stem cell (Stem), and two stromal cell types (Endothelial cells, Fibroblasts) (Fig. 3A). Next, we examined whether any of the identified clusters contained co-expressing *POU1F1* and *NR5A1.* Such double-positive cells were found exclusively within clusters of PIT-1 and SF-1 cell lineages. In total, we identified 91 *NR5A1/POU1F1* double-positive cells (0.6% of all cells), with 49 located in PIT-1 cluster and 42 in SF-1 cluster (Fig. 3B). Interestingly, *NR5A1/POU1F1* double-positive cells formed a distinct subcluster within SF-1 gonadotroph cell population, whereas in the PIT-1-positive lineage they were dispersed throughout the entire cell population (Fig. 3C).

Additionally, we confirmed the presence of such double-positive cells in publicly available scRNAseq data from the human fetal pituitary (FPG) as well as from mouse and rat pituitary glands. *NR5A1/POU1F1* double-positive cells were observed not only in the human APG (see Fig. 3) but also in the FPG and in both mouse and rat samples (Supplementary Fig. S2). To explore the developmental origin of these double-positive cells, we integrated scRNAseq data from the human APG and FPG. We processed the data to identify cells from the basic pituitary lineages and performed trajectory analysis focused on the cluster of gonadotroph cells. This analysis revealed that double-positive cells form a distinct subcluster within SF-1 cell population (Fig. 3D) and are located at the end of a separate differentiation trajectory (Fig. 3E). Along this trajectory, *POU1F1* expression increases while *NR5A1* expression decreases over time (Fig. 3F).

### Neoplastic and normal somatotroph cells

Intriguingly, *NR5A1/POU1F1* double-positive cells were identified in both PIT-1 and gonadotroph lineages of the normal pituitary gland, whereas RNAseq data from tumors show that double-positive PIT-1/SF-1 tumors are overall more similar to PIT-1–positive rather than gonadotroph PitNETs. To specifically examine pituitary tumor cells, we analyzed publicly available scRNAseq data from somatotroph and gonadotroph PitNETs (25 and 5 samples respectively; Supplementary Table S6).

**Fig. 4:**
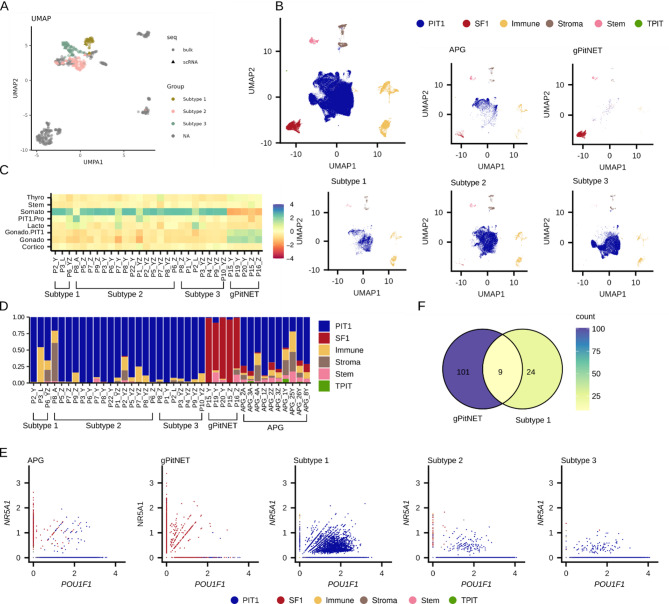
Integrated single-cell RNA-seq data of three subtypes of somatotroph PitNETs (sPitNETs), gonadothroph PitNETs (gPitNETs), and adult pituitary gland (APG). (**A**) UMAP of combined pseudobulk counts of samples from scRNAseq of somatotroph PitNETs and bulk RNAseq data of all somatotroph tumors from the study cohort, colored by three transcriptomic subtypes of sPitNETs. (**B**) UMAP of integrated data from sPitNETs, gPitNETs, and APG with the main cell clusters annotated. (**C**) Similarity of cells in specific samples to cell clusters identified in APG. (**D**) Representation of the main cell clusters across samples. (**E**) Co-expression of *POU1F1* and *NR5A1* with assigned main clusters in specific sample groups. (**F**) Comparison of overlap between genes from the *NR5A1* regulon inferred from gonadotroph cells in gPitNETs and somatotroph cells from Subtype 1 (PIT-1/SF-1–positive) sPitNETs

Each somatotroph tumor was assigned to one of the previously defined transcriptomic subtypes based on classification of their pseudobulk expression profile: three tumors corresponded to Subtype 1 (SF-1/PIT1–positive), 14 to Subtype 2, and eight to Subtype 3 (Fig. 4A). We then integrated scRNAseq data from somatotroph PitNETs with gonadotroph PitNETs and APG, comprising of 40 samples and 142,188 cells. Tumor and non-neoplastic cell populations were grouped into five main clusters (Fig. 4B, Supplementary Fig. S3). Tumor cells from gonadotroph PitNETs closely resembled normal gonadotroph cells, whereas all tumor cells from somatotroph tumors — including the PIT-1/SF-1 double-positive tumors — clustered with normal PIT-1 lineage cells (Fig. 4C). Importantly, tumor cells from double-positive PIT-1/SF-1 somatotroph tumors were clearly unrelated to the *NR5A1*/*POU1F1* double-positive gonadotroph subcluster cells (Gonado.PIT1) observed in normal adult pituitary. Instead, they showed high similarity to other somatotroph tumor cells and to normal PIT-1–lineage cells, and not to gonadotroph tumor cells or normal gonadotrophs, as also evident from the similarity in the expression of key pituitary cell-related genes (Supplementary Fig. S4).

The gonadotroph tumors consisted predominantly of cells of gonadotroph origin, whereas all subtypes of somatotroph tumors were mainly composed of cells of PIT-1 lineage (Fig. 4D). A small number of cells co-expressing *NR5A1* and *POU1F1* was observed in gonadotroph tumors (localized within the SF-1 gonadotroph cell cluster), as well as in two somatotroph tumor subtypes lacking *NR5A1* expression (where these double-positive cells clustered within the PIT-1 cluster) (Fig. 4E).

We also examined the abundance of non-neoplastic cells types — including immune, stromal, and stem-like cells — in individual tumor samples and APG. Notable intertumoral heterogeneous was observed. A high proportion of immune cells was detected in one of three of double-positive (SF-1/PIT-1) somatotroph tumorsamples, which may have influenced the analysis (Fig. 4D). Overall, no consistent differences in the proportions of non-neoplastic cell subtypes were found between different somatotroph tumor subtypes. Compared to APG, somatotroph tumors generally showed lower content of stem-like cells and monocytes, as detailed in Supplementary Fig. S5.

To verify the observation that a distinct set of genes is regulated by SF-1 in double-positive somatotroph and gonadotroph PitNETs, we established regulons of *NR5A1* in gonadotroph tumor cells and double-positive somatotroph tumor cell independently. Analysis of genes within these regulons confirmed the results from the comparison of bulk samples of Subtype 1 and gonadotrhop tumors demonstrating that *NR5A1* regulates different genes in these PitNET types (Fig. 4F, Supplementary Table S7).

**Fig. 5:**
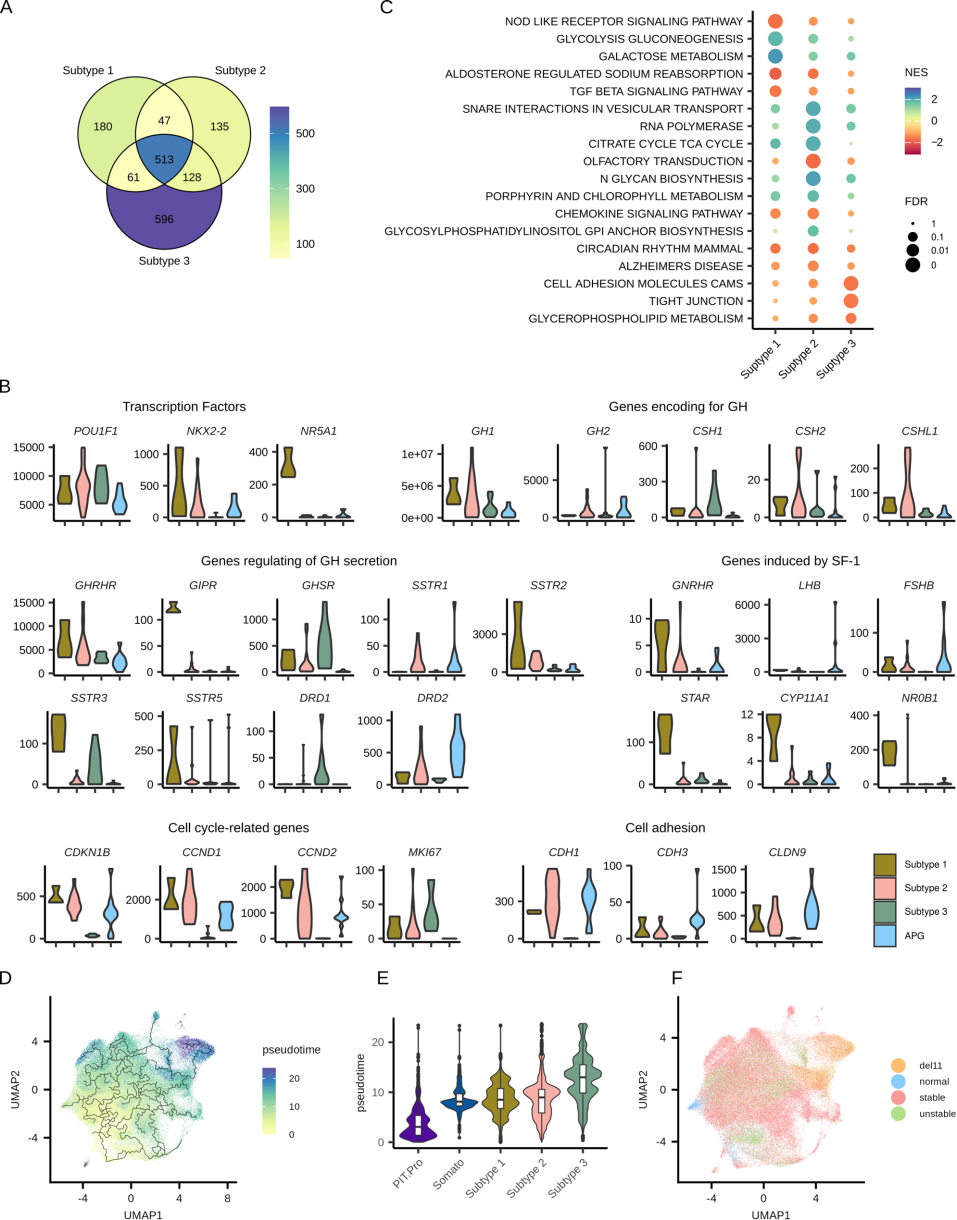
Comparison of tumor somatotroph cells from each transcriptomic subtype of somatotroph PitNETs (sPitNETs) with normal somatotroph cells of the adult pituitary gland (APG). (**A**) Overlap of differentially expressed genes (DEGs) identified in the comparison between tumor somatotroph cells of each transcriptomic subtype of sPitNETs and normal somatotrophs of the APG. (**B**) Expression of selected DEGs in each transcriptomic subtype of sPitNETs and in normal somatotrophs. (**C**) Results of gene set enrichment analysis (GSEA) of DEGs from the comparison of cells from specific transcriptomic subtypes of sPitNETs to APG, based on KEGG database. (**D**) UMAP of somatotroph cells from integrated APG and sPitNET data, colored by pseudotime inferred using Monocle3. (**E**) Distribution of pseudotime across cells from specific cell groups. (**F**) UMAP of somatotroph cells from integrated APG and sPitNET data, classified according to genomic instability as stable, unstable, or with chromosome 11 deletion

Determining abnormal gene expression in somatotroph tumors by comparison with normal pituitary somatotroph cells is not feasible using bulk RNAseq due to the complexed cellular composition of pituitary gland. However, this can be achieved with scRNAseq data. We compared gene expression in tumor cells of each subtype of sPitNETs with normal somatotrophs from the APG. We identified 1,149, 1853 and 2,021 DEGs for Subtypes 1, 2 and 3 respectively, with distinct sets of DEGs unique to each subtype (Fig. 5A, Supplementary Table S8). These results confirmed differences in the expression of key genes related to pituitary function and pathogenesis between subtypes, as well as between tumor and normal cells. Specifically, we observed abnormal expression of TFs (*POU1F1*,* NR5A1*), GH genes (*GH1*,* CSH1*,* CSHL1*), and genes regulating GH secretion (*GHRHR*,* GIPR*,* GHSR*, *SSTRs*, and *DRDs*) (Fig. 5B). Furthermore, the results confirmed an abnormal increase in the expression of SF-1–induced genes in Subtype 1 somatotroph tumors (PIT-1/SF-1–positive), including steroidogenesis-related genes and *GNRHR*, but not *LHB* and *FSHB*. Finally, we observed a tumor-specific decrease in the expression of cell adhesion and cell cycle-related genes in Subtype 3 tumors.

KEGG pathways enriched for the DEGs identified in the comparison between neoplastic cells of each tumor subtype and normal somatotropes were identified (Supplementary Table S9) revealing pathways specific to distinct subtypes (Fig. 5C). Aberrant gene expression was associated with upregulation of galactose and glucose metabolism and downregulation of TGF beta signaling in Subtype 1 tumors; upregulation of TCA cycle in Subtype 2; and downregulation of cell adhesion pathways in Subtype 3.

ScRNAseq data was also used to compute psudotime trajectory of PIT.Pro and somatotroph cells from the APG, alongside somatotroph tumor cells, to explore differences between tumor samples of known subtypes (Fig. 5D). We observed notable variation of pseudotime among cells from specific subtypes, without a clear relationship to the developmental trajectory of normal pituitary cells. However, individual samples from Subtype 3 appeared most distant from normal somatotroph (Fig. 5E). Copy number profiles inferred from scRNAseq data revealed loss of chromosome 11 in these samples (Fig. 5F, Supplementary Fig. S5), a chromosomal loss previously identified as recurrent CNV in Subtype 3 tumors [4, 6].

## Discussion

The classification of pituitary tumors is based on determining from which developmental pituitary lineages they originate [50]. The corticotroph cell lineage is characterized by the expression of TPIT transcription factor, the gonadotroph lineage by SF-1 transcription factor, and the PIT-1-positive lineage includes somatotrophs, lactotrophs and tyreotrophs. In general, PitNETs are categorized into these three main categories: —TPIT, SF-1 and PIT-1 tumors. Tumors arising from distinct pituitary cell lineages that exhibit distinct molecular profiles as observed at the transcriptomic, epigenetic and proteomic levels [14, 18, 20]. However, PitNETs of a particular type still display a degree of heterogeneity in their clinical, histological and molecular features.

Clinically functioning somatotroph pituitary tumors fall into three distinct molecular subtypes, which are not clearly distinguished in the current WHO classification [4–7]. One of these subtypes is characterized by the expression of *NR5A1* (SF-1) transcription factor. Such double-positive PIT-1 and SF-1–expressing sPitNETs have also been occasionally reported by others [8–14] and highlighted as a topic of interest. The preliminary goal of our study was to identify the tumors of this subtype in each publicly available RNAseq dataset from sPitNETs. Indeed, using unsupervised transcriptomic analysis of 193 tumor samples, we found that approximately 20% of sPitNETs are double-positive tumors, confirming their previously observed frequency among acromegaly-associated tumors. Importantly, they were detected in each of five datasets analyzed in this study, which included diverse patient populations (Asian, European, South American).

The developmental identity of tumors co-expressing PIT-1 and SF-1 has been recently discussed in the literature, with various terms used to describe them, including multilineage pituitary [15, 16, 49, 51, 52], gonado/somatotroph [6], somatogonadotroph [17], and plurihormonal tumors [8, 10, 53]. We addressed the question whether these acromegaly-associated sPitNETs truly exhibit molecular features of pituitary gonadotropes and to what extent they resemble SF-1 tumors. Our analysis of 546 pituitary tumor samples revealed that, in terms of gene expression, double-positive sPitNETs are more closely related to other PIT-1 tumors than to gonadotroph tumors. Using scRNAseq data, we compared tumor cells from each subtype of somatotroph tumors and gonadotroph PitNETs with normal pituitary cells of both PIT-1 and SF-1 lineages. This analysis clearly confirmed that double-positive Subtype 1 sPitNETs are counterparts of normal PIT-1 cells, not pituitary gonadotropes. The results of comparing the activity of known TFs involved in differentiation of pituitary cell lineages support this observation. PIT-1/SF-1 sPitNETs display activity of TFs known to participate in the development of the PIT-1 lineage, but not SF-1 lineage such as *POU1F1*, *PROP1*, *CEBPD* or *FOXO1* [54]. Aside from SF-1 (*NR5A1*) itself, these tumors do not exhibit activity of other TFs known to be associated with the gonadotroph lineage, such as *GATA2* or *GATA3* [55].

Part of PIT-1/SF-1–positive sPitNETs were found to express LH in immunohistichemical staining [6, 7, 17]. Our analysis confirms that these tumors indeed express both *LHB* and *GNRHR* at levels comparable to gonadotroph tumors. Both of these genes are regulated by the SF-1 transcription factor in pituitary gonadotroph cells [56, 57]. Interestingly, a category of somatotroph pituitary tumors that respond with increased GH secretion to luteinizing hormone‑releasing hormone (LHRH) was recently described [58]. Most of these tumors also exhibited paradoxical increase of GH in response to an oral glucose tolerance test [58], a phenomenon observed in a specific group of patients with acromegaly [59] as a result of ectopic expression of *GIPR* [60]. *GIPR* expression is correlated with *NR5A1* levels in sPitNETs [7] and it corresponds to demethylation at the *GIPR* promoter, which was observed in SF-1/PIT-1–positive sPitNETs [5]. The presented analysis of both bulk- and scRNAseq data confirms that these double-positive sPitNETs are the ones that express high levels of *GIPR* and expression of this gene distinguishes the tumor cells of this subtype from normal pituitary somatotrophs, which are GIPR-negative. These tumors also express *LHB* and *GNRHR*; therefore, we conclude that Subtype 1 sPitNETs are those that increase GH secretion after LHRH stimulation or glucose load.

Since the expression of *LHB* and *GNRHR* is related to *NR5A1* in both Subtype 1 sPitNETs and gonadotroph PitNETs, other genes regulate by this TF turn out to be differentially expressed in these tumor types. The best-validated gene positively regulated by SF-1 is *STAR*, which encodes a protein playing a key role in steroidogenesis in adrenal cortex and ovary [61]. We clearly observed high expression of *STAR* in Subtype 1 sPitNETs, but not in gonadotorph tumors. Conversely, other SF-1–regulated genes such as *FSHB*, *CYP11A1*, and *NR0B1* (DAX1) exhibit higher expression levels in gonadotroph tumors. More generally, our analysis of *NR5A1* regulons in bulk and scRNAseq data indicates that this TF regulates a somewhat different set of genes in double-positive sPitNETs than in gonadotropinomas. Furthermore, our regulon analysis confirms a previous report that *NKX2-2* transcription factor can be considered specific to PIT-1/SF-1 sPitNETs [49], as we observed the highest activity of *NKX2-2* in this tumor subtype.

The analysis scRNAseq from normal APG showed the presence of a few PIT-1/SF-1 cells among both PIT-1 and gonadotroph lineage cells. Similarly, such cells were identified in human fetal pituitary as well as in pituitary from mouse and rat. Interestingly, normal PIT-1/SF-1 double-positive cells represent a specific developmental subcluster of gonadotroph cells. However, double-positive tumor cells from Subtype 1 sPitNETs turned out to be completely dissimilar (unrelated) to this gonadotroph cluster. Thus, we conclude that these tumor cells originate from PIT-1 lineage, not from gonadotroph cell subtype.

Taking advantage of the scRNAseq data, we compared genes expression in each subtype of sPitNETs with normal somatotrophs to identify tumor-specific aberrantly expressed gens. The profile of aberrant gene expression in tumor cells of each sPitNET subtype confirms the difference in gene expression observed in the current bulk RNAseq results and previously reported findings [6, 7]. Additionally, by integrating the scRNAseq data from normal pituitary and sPitNETs, we performed a pseudotime analysis to align tumor samples with normal developmental trajectory from ProPit cell to somtotroph lineage. The results show that somatotroph PitNETs are more closely related to mature somtotroph lineage, and no clear differences in developmental trajectory among the three tumor subtypes were observed. An interesting finding from this analysis is that Subtype 3 tumors are the least related to normal somatotrophs and are characterized by chromosome 11 loss. This cytogenetic change has already already been recognized as a recurrent DNA copy number variant in sparsely granulated sPitNETs [4, 6].

In this study, we were able to collect a large data set of bulk RNAseq results from PitNETs, but much smaller number of scRNAseq data are currently available. By combining the data from five studies, we identified only three PIT-1/SF-1 sPitNETs, which should be considered the main limitation of our study. The low number of these samples influences the statistical power of inference, while variability within this group prompts us to interpret the single-cell data with caution. For example, the results suggest increased *SSTR2* expression in this tumor subtype, as previously reported [15], but this observation is notably biased by its very high expression in one out of three samples. The role of immune component of the tumor microenvironment in sPitNET has been described [62], and scRNAseq results allow for an in-depth analysis of the microenvironment cell composition. Unfortunately, given intertumoral heterogeneity, we were not able to draw a clear conclusions on differences in the microenvironment between the three subtypes of sPitNETs.

## Conclusions

From the perspective of transcriptomic profiling, we do not find the rationale for considering PIT-1/SF-1 sPitNETs as multilineage or “somatogonadotoph” tumors. These tumors exhibit a gene expression pattern and transcription factor activity profile very similar to other somatotroph tumors, with no resemblance to gonadotroph tumors apart from the expression of *NR5A1* and certain SF-1-regulated genes such as *LHB* and *GNHRH*. Notably, SF-1 appears to regulate a slightly different set of genes insPitNETs compared to gonadotroph tumors, with *STAR* serving as an example of this difference. Accordingly, the tumor cells of PIT-1/SF-1 sPitNETs are closely related to normal pituitary somatotrophs rather than gonadotrophs, although a subtype of normal gonadotroph cells expressing *POU1F1* was also identified.

## Supplementary Information

Below is the link to the electronic supplementary material.

Supplementary Material 1

Supplementary Material 2

## Data Availability

Freely available datasets from bulkRNAseq (accession numbers: HRA003588, E-MTAB-11889, E-MTAB-7768, GSE213527, GSE209903) and scRNAseq (accession numbers: PRJCA009690, GSE208108, HRA003110, HRA003483, PRJNA1137596 and GSE142653).
